# Death of Professor Post

**Published:** 1828-08

**Authors:** 


4. *Death of Dr. Post.*—This distinguished member of the profession died in the city of New-York, on the 14th of June last, in the 63d year of his age. He had been a professor of Anatomy in his native city, for 32 years—from 1793 to 1825 when he resigned from bad health. As a public teacher, he was much esteemed for his simplicity of manner and his numerous practical facts. The Editors of the New-York Medical and Physical Journal, ascribe to him the enviable distinction of having for 20 years been the 'undisputed head of the profession in that city.' We, ourselves, lately had the pleasure of an interview with the deceased, and were much pleased with his dignity, intelligence and suavity. The Medical Society of New-York, have appointed Dr. John A. Smith, the present professor of Anatomy, in the school to which Dr. P. had belonged, to pronounce his eulogy.

